# Impact of the COVID-19 pandemic on sex-related disparities in cystic fibrosis healthcare utilization and outcomes: A population study

**DOI:** 10.1371/journal.pone.0345009

**Published:** 2026-05-06

**Authors:** Jason Weatherald, Chuan Wen, Heather Sharpe, Winnie M. Leung, Paul E. Ronksley, Jeffrey A. Bakal, Michael K. Stickland, Douglas P. Gross, Grace Y. Lam

**Affiliations:** 1 Division of Pulmonary Medicine, Department of Medicine, University of Alberta and Alberta Health Services, Edmonton, Alberta, Canada; 2 Alberta Respiratory Centre, University of Alberta, Edmonton, Alberta, Canada; 3 Provincial Research Data Services, Alberta Health Services, Edmonton, Alberta, Canada; 4 Alberta Strategy for Patient Oriented Research Unit (ASPORU), Edmonton, Alberta, Canada; 5 Medicine Strategic Clinical Network – Respiratory Health Section, Alberta Health Services, Edmonton, Alberta, Canada; 6 Department of Community Health Sciences, Cumming School of Medicine, University of Calgary, Calgary, Alberta, Canada; 7 O’Brien Institute for Public Health, University of Calgary, Calgary, Alberta, Canada; 8 Department of Physical Therapy, University of Alberta, Edmonton, Canada; 9 Women and Children’s Health Research Institute, University of Alberta, Edmonton, Alberta, Canada; University of Colombo Faculty of Medicine, SRI LANKA

## Abstract

**Background:**

The COVID-19 pandemic disrupted healthcare utilization and access for many, with persons living with pre-existing pulmonary conditions like cystic fibrosis (pwCF) having been the most impacted. PwCF appeared to have improvements in lung function and reduction in pulmonary exacerbations during the pandemic. However, it is not clear if general healthcare utilization beyond CF centers were also reduced and whether there existed any sex-based differences in health outcomes during the pandemic. Our objective was to use population-level administrative data to gain a comprehensive understanding of healthcare utilization and outcomes for pwCF pre- compared to post-COVID.

**Methods:**

A retrospective provincial-level analysis was conducted using linked administrative datasets from a single-payer health jurisdiction in Alberta, Canada. We measured hospitalization, emergency department and outpatient visits in pwCF 18 months before and after March 12, 2020. Subgroup analysis was undertaken to differences between sexes.

**Results:**

Acute care encounters (including general emergency department [ED] visits and hospitalizations) for pwCF declined during the pandemic. There was a trend towards an increase in outpatient primary care and specialist clinics (both virtual and in-person) in the post-COVID period. Mortality rate was largely unchanged during the pandemic. CF females and males experienced the same relative change in healthcare utilization during the pandemic with a greater reduction in ED visits by CF females.

**Conclusion:**

PwCF accessed acute care resources less but females experienced a greater drop in ED visits despite similar hospitalization rates as males, raising the possibility that females with CF experienced a disproportionate barrier to accessing acute care.

## Introduction

Healthcare delivery was greatly impacted by the COVID-19 global pandemic. The impact of public health measures may have had a direct impact on clinical outcomes for individuals living with cystic fibrosis (CF). Respiratory-related viruses tend to spark concern for persons with cystic fibrosis (pwCF), as they often exacerbate symptoms. Early reports indicated worse outcomes for vulnerable individuals such as those with co-morbidities [1]. However, PwCF are generally younger with a lower prevalence of obesity historically, which are risk factors for more severe outcomes post COVID-19 infection. Early reports showed PwCF were not at risk for more severe acute COVID-19 outcomes despite having a chronic lung disease that reduced lung function, although healthcare delivery may have been altered [1–3]. Interestingly, COVID-19 incidence in pwCF appeared to be lower than the general population, although some sub-groups (such as those that have a history of organ transplantation) may develop more severe disease [4,5].

Unfortunately, less information exists about the wider impacts of care alterations necessitated by the pandemic on CF healthcare utilization and mortality outcomes. Telehealth and remote monitoring became a common tool for outpatient follow-up in pwCF to reduce risk of viral exposure [6,7]. Existing data from pre-public availability of elexacaftor/tezacaftor/ivacaftor (ETI) from other high-income countries demonstrated improvements in lung function for both adults and children, including a reduction in pulmonary exacerbations paired with a dramatic rise in use of telehealth outpatient visits [7]. This suggests that pulmonary outcomes may have improved during the pandemic, possibly due to enhanced social isolation practices or heightened infection control measures [4,8,9]. It remains unclear whether the reduction in exacerbations were the result of improved health or care avoidance, as the improvements noted in lung function may be biased by those who have access to home spirometry or were motivated to attend the pulmonary function labs for testing. Importantly, there exists a well-recognized sex-based disparity in CF outcomes that adversely impacts females [10], with systemic barriers arising during the pandemic that disproportionately impacted females more than males [11]. Thus, it is unclear what health care utilization and mortality outcomes were for pwCF during the pandemic or whether sex-based differences existed. Based on existing literature, we hypothesized that the reduction in pulmonary exacerbations reported would lead to a reduction in general acute care utilization, a rise in (predominantly virtual) outpatient visits and a reduction in mortality during the pandemic, in both females and males. The objective of this study is to describe general healthcare utilization and mortality of pwCF using population-level administrative data from a public funder of health services in Canada and to see if these measures differed by sex.

## Study design and methods

### Study design and case definitions

This retrospective cohort study was completed in the province of Alberta, Canada (estimated 2020 population of 4.4 million). The study compared 18-month periods before (pre-COVID) and after (post-COVID) the day the World Health Organization (WHO) declared a COVID-19 global pandemic (March 12, 2020). Cases of adult CF (aged 18 years and older) were identified using the following case definition: ≥ 1 inpatient practitioner claim OR ≥2 outpatient claims at least 30 days apart using ICD-9 or ICD-9-CM code of 277.0x or ICD-10 code of E84.[12,13] Post-lung transplant cases were defined as ≥1 practitioner claim for ICD-9 code V42.6 (outpatient) or ICD-10 code of Z94.2 (inpatient) [12,13]. Prevalent cases were defined by a cohort entry date prior to September 12, 2018, which marks the beginning of the pre-COVID period. The survived prevalent cohort was defined as the pre-COVID prevalent cases of CF who were alive as of March 12, 2020. The study was conducted in accordance with ethical guidelines, complies with RECORD reporting guidelines [14] for the use of secondary data sources, and approved by the University of Alberta Research Ethics Board (Pro00119420).

### Data sources

Provincial-level administrative health data sources from a public funder of health services were linked for use in this study. This included the National Ambulatory Care Reporting System (NACRS), Inpatient Discharge Abstract Database (DAD), Practitioner Claims (CLAIMS), Pharmaceutical Information Network (PIN), Alberta Health Services Enterprise Data Warehouse (EDW) schema labeled “COVID-19” (for COVID-19 test results) and Alberta Population Registry. Personal health numbers were used to complete the data linkages. We extracted comprehensive data on hospitalizations, emergency department (ED) visits, outpatient clinic (including virtual and in-person; specialists and general practitioner) appointments (restricting claims to ‘community’ only), physician billing claims, COVID-19 testing, and neighborhood-level census data. We limited outpatient primary care visits to one primary care provider per patient per day. Biological sex recorded in the Alberta Health Care Insurance Plan (AHCIP) Registry was used to determine sex as male, female or unknown. There were no individuals who were identified as ‘unknown sex’ in the cohort.

### Primary outcomes measures and definitions

We assessed hospitalizations, ED visits, general and outpatient primary care/specialist visits and all-cause mortality, which were compared among the CF prevalent and survived CF prevalent pre- and post-COVID using physician claims data. Virtual visits were defined as community physician claims that contained specific Canadian Classification of Procedures Extended codes for virtual care. All outpatient visits were defined as any community-based physician visits occurring during the study observation period. To capture the full burden of care, visits were counted based on unique provider identifiers per day. In person visits were determined by subtracting all outpatient visits minus virtual visits. For each outcome, we reported both the aggregate count of events and the total number of affected individuals. We also generated binary indicators to note the occurrence or non-occurrence of each outcome. For all-cause mortality, we defined pre-COVID death as death prior to March 12, 2020. COVID-19 antigen testing was determined by health records. Home rapid antigen tests were not widely available in the province during the study period and therefore were not included in the determination of COVID-19 infection.

### Comorbidities

In assessing comorbidities for mortality analysis, we utilized the Charlson Comorbidity Index (CCI) by combining data from the DAD, NACRS, and CLAIMS databases.[15] The selected data covered a two-year interval before both pre-COVID (starting September 12, 2018) and post-COVID (starting March 12, 2020) study durations. 5.8% of material deprivation index data was missing. A sensitivity analysis was done, which demonstrated no meaningful differences after exclusion of these cases.

### Statistical analysis

For baseline retrospective cohort comparisons, we employed Fisher’s exact/chi-squared tests and Mann-Whitney/Wilcoxon rank-sum test as appropriate. Given the evidence of overdispersion in our data (overdispersion was assessed with a standard Poisson model), we utilized negative binomial regression models for the incidence rate ratio (IRR) of hospitalization, and ED and outpatient visits. To calculate the adjusted HR (adj-HR) and IRR, we included age, sex, CF duration, rural-residency status, income and material deprivation index as covariates (derived from Census data) to successfully correct. The Charlson comorbidity score was additionally included to determine adj-HR. We further undertook stratified analyses based on sex. The modelling included the same covariates listed above minus sex. No person-time offsets were needed as the focus was on survived CF cases. The observation window was fixed for all cases (1.5 years pre and post pandemic). The duration of CF was not included in the final model. Statistical significance was defined as *p* < 0.05. All statistical analyses were conducted using SAS v.9.4 (Cary, N.C.).

## Results

### Baseline demographics

**Fig 1:**
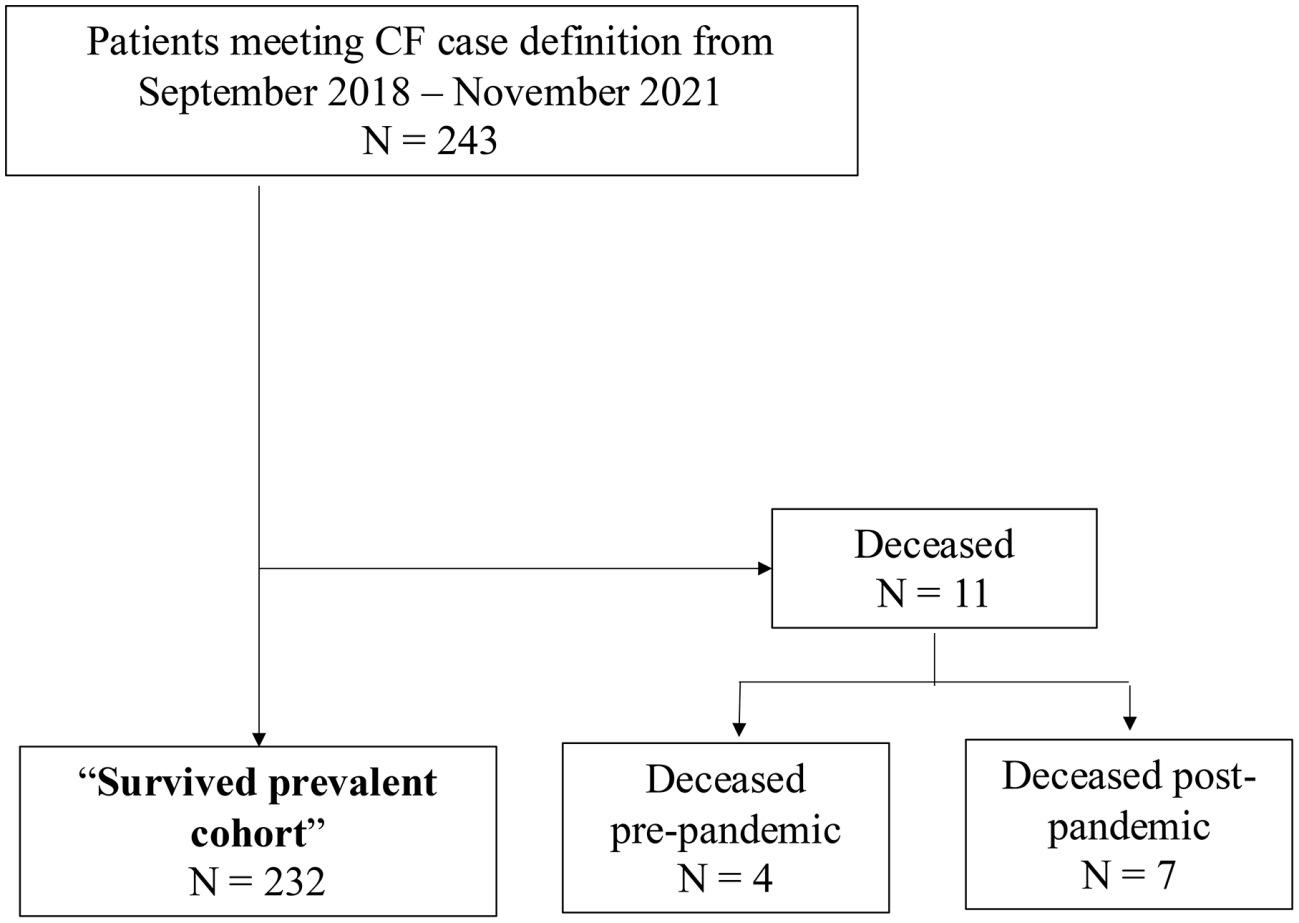
Flow diagram depicting the membership of the survived prevalent cohort used in this study.

There were 243 unique pwCF identified in the entire 36 months’ study period (Table 1) with a slight female preponderance (52.7%). There were 11 deaths during the study period (4 died pre-COVID and 7 post-COVID), resulting in 232 cases for analysis (termed the survived prevalent cohort; Fig 1). PwCF that survived the pandemic were significantly younger (mean (SD): 35.7y (14.8) vs 60.5y (25.4), p < 0.0001). Our data shows a statistically significant difference in CCI levels between the survived and deceased groups (p = 0.003). Specifically, compared to the survived group, the deceased group had a much higher proportion of cases with a high CCI (18.2% vs. 0.9%). There were no significant differences in age by sex, rural/urban residency, income or material deprivation index.

**Table 1:** Clinic demographics of the study cohort (N = 243).

	Total	Survived, n (%)	Deceased, n (%)	p value	Female, n (%)	Male, n (%)	p value
**Sex**	243	232	11	/	128	115	/
Female, n (%)	128 (52.7)	121 (52.2)	7 (63.6)	0.5	/	/	/
Male, n (%)	115 (47.3)	111 (47.8)	4 (36.4)		/	/	/
**Age (years), mean (SD)**	36.8 (16.2)	35.7 (14.8)	60.5 (25.4)	<0.0001	36.2 (16.5)	37.5 (15.9)	0.5
**Median (IQR)**	32 (24, 46)	32 (24, 45)	70 (33, 82)	0.001	31 (24, 46)	34 (26, 46)	0.3
**Rural Residency**	45 (18.5)	44 (19.0)	1 (9.1)	0.7*	26 (20.3)	19 (16.5)	0.4
**Geographic zone, n (%)**				0.8*			0.03
South	20 (8.2)	20 (8.6)	0 (0.0)		13 (10.2)	7 (6.1)	
Calgary	83 (34.2)	77 (33.2)	6 (54.5)		43 (33.6)	40 (34.8)	
Central	38 (15.6)	37 (15.9)	1 (9.1)		14 (10.9)	24 (20.9)	
Edmonton	77 (31.7)	74 (31.9)	3 (27.3)		39 (30.5)	38 (33.0)	
North	25 (10.3)	24 (10.3)	1 (9.1)		19 (14.8)	6 (5.2)	
**Income (IQR)**	52,891 (44,464, 64,141)	54,802 (46,263, 80,656)	53,538 (45,340, 65,392)	0.4	53,364 (44,658, 67,411)	53,806 (46,367, 62,896)	0.6
**Material Deprivation Index**				0.3			0.1
Quintile 1 (Most privileged)	41 (16.9)	39 (16.8)	2 (18.2)		25 (19.5)	16 (13.9)	
Quintile 2	43 (17.7)	43 (18.5)	0 (0.0)		18 (14.1)	25 (21.7)	
Quintile 3	55 (22.6)	52 (22.4)	3 (27.3)		28 (21.9)	27 (23.5)	
Quintile 4	52 (21.4)	50 (21.6)	2 (18.2)		24 (18.8)	28 (24.3)	
Quintile 5 (Most deprived)	38 (15.6)	34 (14.7)	4 (36.4)		22 (17.2)	16 (13.9)	
Missing/not recorded	14 (5.8)	14 (6.0)	0 (0.0)		11 (8.6)	3 (2.6)	
**Charlson Comorbidity Index**				0.003*			0.03*
0-2	234 (96.3)	226 (97.4)	8 (72.7)		126 (98.4)	108 (93.9)	
3-4	5 (2.1)	4 (1.7)	1 (9.1)		0 (0.0)	5 (4.3)	
>=5	4 (1.6)	2 (0.9)	2 (18.2)		2 (1.6)	2 (1.7)	
**Death**							
Pre-covid	4	/	4		3 (75)	1 (25)	
Post-covid	7	/	7		4 (57.1)	3 (42.9)	
**No death**	232	232	/		121 (52.2)	111 (47.8)	
**Covid-19 test**							
Negative (%)	146	143 (97.9)	3 (2.1)	0.2*	75 (51.4)	71 (48.6)	1.0*
Positive (%)	6	5 (83.5)	1 (16.5)		3 (50)	3 (50)	

*Fisher’s test was applied for cases less than 5.

Sex-based analysis demonstrated that the baseline demographics between females and males were largely similar with regard to age, rural residency, income or material deprivation index (Table 1). However, females were more likely to have a lower Charlson Index or fewer comorbidities (p = 0.033). No other clinically relevant differences were identified.

### Healthcare utilization

There was a reduction in general ED visits from pre-COVID compared to post-COVID by PwCF (adjusted incident rate ratio (adj-IRR) 0.55; 95% CI 0.40, 0.75; < 0.001) (Table 2). This reduction in adj-IRR of ED visits during the pandemic was similarly seen in both males (0.58; 95% CI 0.36, 0.54; p = 0.03) and, to a greater extent, females (0.48; 95% CI 0.30, 0.75, p < 0.001) (Table 2). The crude ED rate was pre-COVID 1,942.5 per 1,000 person-years; post-COVID was 1,020.1 per 1,000 person-years. Adj-IRR for general hospitalizations was also reduced from pre-COVID compared to post-COVID (0.64; 95% CI 0.46, 0.89; p = 0.008). However, this change was not reflected in the sex based analysis (females 0.69; 95% CI 0.46, 1.05; males 0.63; 95% CI 0.36, 1.07) (Table 3). Using sex as an interaction term in the regression modelling revealed that sex was not a significant predictor of general hospitalizations (0.66 (0.42, 1.03), p = 0.068). The crude hospitalization rate was pre-COVID 706.9 per 1,000 person-years; post-COVID 456.9 per 1,000 person-years.

**Table 2:** ED visit Pre-and Post-pandemic in the CF survived prevalent Cohort Analyzed by sex.

	Total (n = 232)^#^			Female (n = 121)^$^			Male (n = 111)^$^		
	Pre COVID	Post COVID	p value	Pre COVID	Post COVID	p value	Pre COVID	Post COVID	p value
**Number of cases with ≥1 ED visits, n (%)**	136	112	0.03	80	56	0.002	56	56	1.0
**Number of ED visits, n**	676	355	0.01	406	190	0.004	279	165	0.6
**Crude IRR, (95% CI)**	0.51 (0.37, 0.71)		<0.001	0.47 (0.3, 0.73)		<0.001	0.61 (0.38, 0.99)		0.04
**Adjusted IRR, (95% CI)**	0.55 (0.40, 0.75)		<0.001	0.48 (0.30, 0.75)		0.001	0.58 (0.36, 0.94)		0.03

IRR: Incidence rate ratio; CI: confidence interval.

# Adjusted for age, sex, rural residency, Charlson comorbidity index (negative binomial model).

$ Adjusted for age, rural residency, Charlson comorbidity index (negative binomial model).

**Table 3:** All hospitalization pre-and post-pandemic in the CF survived prevalent Cohort Analyzed by sex.

	Total (n = 232)^#^			Female (n = 121)^$^			Male (n = 111)^$^		
	Pre COVID	Post COVID	p value	Pre COVID	Post COVID	p value	Pre COVID	Post COVID	p value
**Number of cases with ≥1 all hospitalization, n (%)**	102	74	0.007	62	42	0.009	40	32	0.3
**Number of all hospitalization, n**	246	159	0.005	140	93	0.01	106	66	0.2
**Crude IRR, (95% CI)**	0.65 (0.46, 0.9)		0.01	0.66 (0.44, 1.0)		0.05	0.63 (0.36, 1.07)		0.09
**Adjusted IRR, (95% CI)**	0.64 (0.46, 0.89)		0.008	0.69 (0.46, 1.05)		0.08	0.63 (0.36, 1.07)		0.09

IRR: Incidence rate ratio; CI: confidence interval.

# Adjusted for age, sex, rural residency, Charlson comorbidity index (negative binomial model).

$ Adjusted for age, rural residency, Charlson comorbidity index (negative binomial model).

There were no significant differences in outpatient visits pre-COVID compared to post-COVID regardless of sex, though there is a trend towards an increase in outpatient visits in the post-COVID period (Table 4). The outpatient visit crude rate was pre-COVID 13,158.0 per 1,000 person-years and post-COVID 14,797.4 per 1,000 person-years. Further subgroup analysis into general practitioner versus specialist (which includes CF clinic visits) revealed no significant differences pre- compared to post-COVID regardless of sex when data are examined by total events or event rate (Table 5).

**Table 4:** All outpatient visit Pre-and Post-pandemic in the CF survived prevalent Cohort Analyzed by sex.

	Total (n = 232)^#^			Female (n = 121)^$^			Male (n = 111)^$^		
	Pre COVID	Post COVID	p value	Pre COVID	Post COVID	p value	Pre COVID	Post COVID	p value
**Number of cases with ≥1 all outpatient visits, n (%)**	232	231	0.3	121 (100)	121 (100)	1.0	111 (100)	110 (99.1)	0.3
**Number of all outpatient visits, n**	4579	5146	0.3	2650	3002	0.225	1929	2144	0.9
**Crude IRR, (95% CI)**	1.12 (1.0, 1.27)		0.06	1.13 (0.97, 1.32)		0.118	1.11 (0.93, 1.33)		0.3
**Adjusted IRR, (95% CI)**	1.10 (0.97, 1.23)		0.1	1.10 (0.94, 1.28)		0.235	1.11 (0.93, 1.32)		0.3

IRR: Incidence rate ratio; CI: confidence interval.

# Adjusted for age, sex, rural residency, Charlson comorbidity index (negative binomial model).

$ Adjusted for age, rural residency, Charlson comorbidity index (negative binomial model).

**Table 5:** Outpatient visits (survived cohort) split by General Practitioner versus Specialist visits during the Pre- versus Post-COVID periods.

	Events, n						Event rate, avg visits per individual					
	Female			Male			Female			Male		
	Pre COVID	Post COVID	P value	Pre COVID	Post COVID	P value	Pre COVID	Post COVID	P value	Pre COVID	Post COVID	P value
**All visit**	2650	3002		1929	2144		21.9	24.8		17.4	19.5	
**General** **Practitioner**	638	695	0.432	563	588	0.2	6.4	7.4	0.9	7.5	8.1	0.8
**Specialist**	2012	2307		1366	1556		16.6	19.1		12.3	14.4	

Generalized Estimating Equation (GEE) modelling was conducted for healthcare utilization outcomes, including period (post- vs pre-COVID), sex, and a sex-by-period interaction, adjusting for age, residency (urban vs rural), and Charlson comorbidity score. (Table 6). Across all three outcomes, the sex-by-period interaction was not statistically significant, ED visits interaction (p = 0.21), hospitalizations interaction (p = 0.82), outpatient visits interaction (p = 0.94).

**Table 6:** Generalized Estimating Equation (GEE) Model Results for Healthcare Utilization Outcomes Testing Sex-by-Period Interactions.

Outcomes	Estimate (β)	95% Confidence Limits	P-value
**Emergency Department (ED) Visits**			
**Post-COVID vs Pre**	−0.7991	(−1.33, −0.26)	**0.003**
**Sex (Male vs Female)**	−0.3563	(−1.06, 0.34)	0.3
Interaction: Post-COVID * Sex (Male)	0.3825	(−0.22, 0.98)	0.2
**Age**	0.0053	(−0.021, 0.031)	0.7
**Residency (Urban vs Rural)**	1.0946	(0.58, 1.61)	**<0.0001**
**Charlson Score**	0.0953	(−0.072, 0.26)	0.3
**All-Cause Hospitalizations**			
**Post-COVID vs Pre**	−0.4537	(−0.75, −0.16)	**0.003**
**Sex (Male vs Female)**	−0.1889	(−0.61, 0.23)	0.4
Interaction: Post-COVID * Sex (Male)	−0.0563	(−0.54, 0.43)	0.8
**Age**	0.0038	(−0.011, 0.019)	0.6
**Residency (Urban vs Rural)**	0.6894	(0.29, 1.08)	**0.0006**
**Charlson Score**	0.0087	(−0.24, 0.26)	0.9
**Outpatient Visits**			
**Post-COVID vs Pre**	0.1097	(0.016, 0.20)	**0.02**
**Sex (Male vs Female)**	−0.246	(−0.42, −0.077)	**0.004**
Interaction: Post-COVID * Sex (Male)	−0.0068	(−0.18, 0.17)	0.9
**Age**	0.0036	(−0.0021, 0.0093)	0.2
**Residency (Urban vs Rural)**	−0.0389	(−0.25, 0.17)	0.7
**Charlson Score**	0.0393	(−0.053, 0.13)	0.4

### All-cause mortality

Of the 4 deaths pre-COVID, 3 were female and 1 was male; while the 7 post-COVID deaths were split between 4 females and 3 males (Table 1). A total of 152 individuals underwent lab-reported COVID-19 testing during the post-COVID period, of which, 6 individuals tested positive (Table 1).

## Discussion

This study of population-level administration data explored health care utilization and mortality of pwCF during the COVID-19 pandemic. It is important to note that the data for this study was pre-ETI public availability in the province. Several registry studies have provided reassuring insights into the overall maintenance of health outcomes during the pandemic in the CF population. Our study builds upon these existing findings of registry data by examining the wider healthcare utilization pattern of this patient population with regard to care provided beyond the CF clinic to determine if there was a difference in general healthcare utilization between sexes. We found that overall, similar to published data on CF-specific care utilization, general hospitalizations and ED visits decreased during the pandemic. Outpatient visits to primary and specialists (i.e., CF clinics) did not change, although there was a trend towards increased visits during the pandemic. This trend of reduced acute care utilization with a compensatory trend of increased outpatient visits has been demonstrated in other chronic pulmonary small airways disease, including COPD and asthma [16,17].

Early public health controls implemented to reduce the transmission of COVID-19 may have been beneficial for pwCF, as these enhanced infection control measures may have reduced the spread of communicable respiratory infections, the main driver of CF exacerbations [4,8,9]. Consistent with this theory, unadjusted all-cause mortality rates we observed did not significantly differ pre- compared to post-COVID. Of the pwCF that died, they were significantly older than those that survived and had more comorbidities. While these numbers were too small to allow for any meaningful statistical analyses (4 versus 7 deaths pre- compared to post-COVID), we suspect the consistent rate of mortality likely reflects the natural course of CF, rather than the pandemic. Sex based analysis showed that the pandemic experience was largely similar between females and males, though females had a greater reduction in ED visits during the pandemic (Table 2). It is unclear whether this is the result of better protection from infection (and therefore fewer exacerbations) during the pandemic, given that females were shown to have better compliance with COVID-19 public health measures [18]. It may also reflect the greater barriers to health access and delayed seeking health care that disproportionately impacted females over males [11,19]. We postulate that because the relative reduction in hospitalization pre- compared to post-COVID was similar in both sexes (Table 3), it is plausible that the outsized reduction in ED visits for CF females was driven at least in part by barriers to acute care access rather than improvements in health. However, other biologic factors might also have contributed to this difference, which are beyond the scope of this study.

The observed reduction in pwCF requiring hospitalization during the pandemic was unexpectedly lost when the data were examined in sex-based subgroup analysis (Table 3). We hypothesize that the sex-based analysis resulted in a loss of statistical power to identify differences in a smaller population. We tried to address this possibility by running additional analysis using sex as an interaction term and found that sex was not a predictor of hospitalization. Therefore, taken together, the reduction in hospitalization during the post-COVID timeframe was not explainable by sex. This further contributes to a possible hypothesis that CF females likely did not have better health than males to account for the relatively greater reduction in ED visits.

There are several limitations of this study that should be noted. First, this study was predicated on administrative data, and as such, we did not have access to more granular patient-level clinical data such as forced expiratory volume in one second (FEV_1_) or body mass index (BMI) to determine current disease severity. However, as studies from other high-income countries have provided this information, our study adds to this existing literature with additional data outside of registry-based information, taking a comprehensive look at all healthcare utilization by pwCF. Second, the prevalence of CF on a population level is relatively small, which poses some limitations on subgroup analyses due to reduced statistical power and precision, particularly for mortality outcomes. Third, given that this is a retrospective administrative database study, our findings are subject to survivor bias (i.e., only those who were healthy enough to survive to the start of the COVID-19 pandemic were included in the study). Thus, the healthcare utilization data may be enriched for individuals who had milder CF or other comorbidities. Fourth, our study focused on the first 18 months of the pandemic. As COVID-19 variants, vaccination availability and public health policies evolved, we anticipate that the experience over the rest of the pandemic and after might follow a different healthcare utilization trajectory. Additional research to explore changes in healthcare utilization during the rest of the pandemic would be needed to determine if there was a “rebound” phase of care-seeking behaviour within the pwCF population. Finally, the accessible provincial mortality database primarily collects all-cause mortality data. As a result, we cannot directly determine rates of CF-related deaths.

### Interpretation and conclusion

This study demonstrated that pwCF had less acute care utilization (hospitalizations and ED visits) during the pandemic for females and males, with a trend towards increased outpatient care utilization. Mortality was largely unchanged during the pandemic, and is unlikely to be significantly attributable to the pandemic. Further investigation, with a larger sample is required to assess whether the observed changes in healthcare utilization are the result of improved health (due to improved public health measures resulting in decreased respiratory virus transmission), or attributable to avoidance of health care facilities/care access barriers that disproportionately impacted females. Additional examination of healthcare utilization over the rest of the pandemic by pwCF would be illuminating for resource planning of future pandemics.

## Abbreviations

CI: confidence interval
ED: emergency department
IRR: incidence rate ratio
adj-IRR: adjusted IRR
SD: standard deviation
pwCF: persons with cystic fibrosis
CF: cystic fibrosis
